# Hand-book of Practical Medicine

**Published:** 1887-03

**Authors:** W. E. Casselberry


HANDBOOK OF PRACTICAL MEDICINE. *By* DR. HERMANN EICHHORST, *Professor of Special Pathology and Therapeutics and Director of the University Medical Clinic in Zurich. Volume I. Diseases of the Circulatory and Respiratory Apparatus. One hundred and three wood engravings; pp. v, 401. New York:* WILLIAM WOOD & COMPANY. *Chicago:* W. T. KEENER.

This work of four volumes, which constitutes a portion of Wood's Library for 1886, is a translation under another name of Eichhorst's *Speciellen Pathologie und Therapie*, 1885, although no mention of this fact or of the name of the translator is found on the title page.

It is a most acceptable addition to our literature in English from the German school of medicine. Volume I, to which this notice especially refers, includes the diseases of the circulatory and respiratory apparatus.

The section devoted to cardiac affections is particularly creditable. Even valulan lesions of the right side of the heart, which are ordinarily but superficially considered, receive extended notice. True, pulmonary insufficiency and stenosis are rare, and although relative insufficiency of the tricuspid valve is more frequent, particularly in the course of mitral or aortic disease and in pulmonary affections, isolated tricuspid lesions are also rare. "Among two hundred and thirty cases of valvular lesion, von Bamberger found the tricuspids affected [primarily] only in two." Nevertheless, cases of doubtful character are continually arising, in which it is necessary either to establish or positively to exclude these right-sided valvular defects, and for this purpose a knowledge of their murmurs and paths of conduction is essential.

To diseases of the nasal cavity are apportioned but five pages, and diseases of the larynx fare only a trifle better. The subjects should rather have been omitted entirely, as the purpose of conforming the contents of a book absolutely to its title is hardly sufficient cause for the insertion of chapters scarcely approaching mediocrity into an otherwise laudable work.

Diseases of the lungs are comprehensively treated. Pulmonary tuberculosis, however, is here omitted, evidently being embraced elsewhere in the work under the general subject of tuberculosis.

Regarding the etiology of acute fibrinous or croupous pneumonia, the positive opinion is advanced that "it is an infectious disease, whether it develops primarily or secondarily, and that catching cold possesses a causal significance, only in so far as it favors the occurrence of infection." It is further opined that the disease may be produced, not alone by a single kind of microbe, but by various forms of bacilli. From this standpoint is explained the occurrence of secondary pneumonia during the course of other infectious diseases, *e. g.*, the "schizomycetes" of typhoid fever, gaining access to the alveolar spaces of the lungs, may there occasion a fibrinous pneumonia. Much reason is advanced in support of this argument, and yet it were difficult to conceive, in accordance therewith, why a robust steamship captain, havng been exposed on the steamer-bridge in mid-ocean to a severe gale for forty-eight hours, should suddenly be taken ill and die of pneumonia.

Whilst recognizing the infectious character of the disease in general, in explanation of all cases, it seems to us necessary to assume either that cold may at times serve as the sole etiological factor or that there are two diseases anatomically apparently identical, one an infectious systemic disease with a local manifestation, and the other a simple local inflammatory affection, accompanied by systemic symptoms.

The author recognizes, in a pathological sense, the frequent transition of acute fibrinous pneumonia into chronic interstitial pneumonia or fibroid phthisis, and suggests that chronic bronchitis, broncho-pneumonia, and acute pleuritis, on account of intimate lymphatic communication with the interlobular connective tissue, may also serve as primary causes of fibroid phthisis. This view conforms to practical clinical experience, and the attempt to draw undeviating lines of demarcation between these various affections should therefore cease.

The illustration of the oldest form of pneumatic cabinet, that of Tabarie, is of interest in connection with the recent introduction in this country of more nearly perfect forms of this apparatus.

W. E. Casselberry.

